# Succession Patterns of Microbial Composition and Activity following the Diesel Spill in an Urban River

**DOI:** 10.3390/microorganisms11030698

**Published:** 2023-03-08

**Authors:** Ruiyu Yang, Chao Peng, Yuqiu Ye, Yun Tang, Lu Lu

**Affiliations:** 1 College of Life Sciences, China West Normal University, Nanchong 637002, China; 2 Chemical Synthesis and Pollution Control Key Laboratory of Sichuan Province, China West Normal University, Nanchong 637009, China; 3 College of Environmental Science and Engineering, China West Normal University, Nanchong 637009, China

**Keywords:** diesel biodegradation, microbial dynamics, microbial activity, radiotracer assay, river diesel spill

## Abstract

Diesel spills in freshwater systems have adverse impacts on the water quality and the shore wetland. Microbial degradation is the major and ultimate natural mechanism that can clean the diesel from the environment. However, which, and how fast, diesel-degrading microorganisms could degrade spilled diesel has not been well-documented in river water. Using a combination of ^14^C-/^3^H--based radiotracer assays, analytical chemistry, MiSeq sequencing, and simulation-based microcosm incubation approaches, we demonstrated succession patterns of microbial diesel-degrading activities, and bacterial and fungal community compositions. The biodegradation activities of alkanes and polycyclic aromatic hydrocarbons (PAHs) were induced within 24 h after diesel addition, and reached their maximum after incubation for 7 days. Potential diesel-degrading bacteria *Perlucidibaca*, *Acinetobacter*, *Pseudomonas*, *Acidovorax*, and *Aquabacterium* dominated the community initially (day 3 and day 7), but later community structure (day 21) was dominated by bacteria *Ralstonia* and *Planctomyces*. The key early fungi responders were *Aspergillus*, *Mortierella*, and *Phaeoacremonium* by day 7, whereas *Bullera* and *Basidiobolus* dominated the fungal community at day 21. These results directly characterize the rapid response of microbial community to diesel spills, and suggest that the progression of diesel microbial degradation is performed by the cooperative system of the versatile obligate diesel-degrading and some general heterotrophic microorganisms in river diesel spills.

## 1. Introduction

An oil spill is the anthropogenic input of petroleum into the environment, and it could cause great and long-lasting damage to both the environment and the local economy [1]. An oil spill could occur during the whole process of the modern petroleum industry chain, including the production, transportation, and consuming processes [2]. the type of the leaked oil could be crude oil, as well as other petroleum products such as diesel. As one of the most common productions of the petroleum industry, diesels are widely used as fuel not only in the global automotive and tractor fleet, but also in other facilities such as thermal power plants. Generally, major studies of diesel oil spills are associated with the marine environment [3,4], rather than inland rivers. Inland river diesel spills also cause environmental damage by contaminating the fresh water and riparian ecosystem, which can directly increase concerns toward human health [5]. Although great efforts have been taken to prevent diesel fuel spills, the accelerating development of cities inevitably leads to an increased risk of river oil spills due to water-front construction, high-load discharge, and inland shipping [6]. For example, one of the largest inland fuel diesel spills was an accident in a Russian power station in June of 2020, which released about 200 million kilograms of diesel into the Norilsk–Pyasino lake–river ecosystem and was spread by the river flow over a large area of 350 square kilometers. It has been reported that both the surface water and bottom sediments in the catchment of river were polluted with oil products, phenols, and organic matter [7,8]. Current physicochemical technologies are applied as emergency actions to clean up the spilled oil, but, ultimately, a substantial proportion of oil hydrocarbons are biodegraded by oil-degrading microorganisms [9], which use oil hydrocarbons as energy and carbon sources [10,11]. The complete degradation of diesel compounds, which are mainly a mixture of a series of saturated hydrocarbons and aromatic hydrocarbons, usually requires the cometabolism of several highly specialized hydrocarbon-degrading microorganisms [11,12,13,14]. However, the effect of a diesel spill on the activity and community composition of oil-degrading bacteria in river systems remains largely unexplored.

Many hydrocarbon-degrading taxa across different river ecosystems have been identified in surface water with diesel contamination [15,16], For example, the bacterial genera of *Acinetobacter* and *Staphylococcus* populated the diesel-polluted surface water in the Kizilirmak river and Thesjaswini river, respectively, and both have remarkable capabilities to degrade diesel contaminants [17,18]. Fungi-containing hydrocarbon catabolic enzymes also have the potential to biodegrade diesel hydrocarbons [19,20]. The low specificity of many fungal enzymes facilitates them to cometabolize structurally diverse oil compounds [20]. In addition, it has been reported that microorganisms usually respond quickly to the load of oil hydrocarbons, such as the increase in microbial production rate and changes in the community structure [21,22]. The successive pattern of microbial oil-degrading activities and community compositions are also found to be associated with the changing concentrations of dissolved oil compounds in the water [10,14,23]. However, there is little research of the dynamic changes in microbial communities and their hydrocarbon biodegradation activities in the scenarios of river diesel spills.

The Jialing River is the second longest tributary of the Yangtze River, China, and is the main drinking, industrial, and agricultural water supply for the cities along the river. It also supports much river traffic, including passenger ships and sand dredgers, meaning much diesel fuel is used in this region [24]. Oil spills are considered a major threat to the Jialing River ecosystem, where many diesel spills have been documented [25]. This indicates that studies of an oil spill microbial response there are necessary and urgent. However, data about the presence, diversity, and activity of oil-degrading microorganisms in response to diesel fuel in the Jialing River have not been reported. Hence, the study objectives were as follows: (i) to reveal the bacterial and fungal taxa that would be responsible for the compositional shifts over time in the exposure of diesel fuel; and (ii) to probe the microbial oil biodegradation rates as time progress. ^3^H-leucine incorporation tracing method and MiSeq sequencing were used to detect the changes in the activity and structure of microbial communities, respectively. A radiotracer method using ^14^C-hexadecane and ^14^C-napthalene was employed to directly determine microbial hydrocarbon degradation rates. We also performed gas chromatography coupled with mass spectrometry (GC–MS) to track the diesel biodegradation rate in the experimental microcosms.

## 2. Materials and Methods

### 2.1. Water Sampling Procedure

Surface water samples (100 L) were collected from the Nanchong urban section of the Jialing River (106°5′45.88″, 30°46’40.47″). As depicted in Figure S1, the sampling site of the water sample is near to a dock. The boats are powered by diesel engines. Spilled diesel was seen on the decks. Water samples were obtained by filling six 20 L, sterile acid-washed plastic buckets. After sampling, water samples were stored on ice packs in the field (~4 °C), and transported within 2 h to the laboratory where the experiment was conducted immediately. The physicochemical properties of the water sample were determined. The water pH value was 8.0 ± 0.02. The NO_3_^−^, NH_4_^+^, total phosphorus, and dissolved oxygen contents were 1.2 ± 0.03, 2.1 ± 0.04, 0.2 ± 0.02, and 4.9 ± 0.1 mg/L, respectively. The concentrations of total *n*-alkanes and 16 U.S. Environmental Protection Agency (EPA)-listed priority polycyclic aromatic hydrocarbons (PAHs) were 0.1 mg/L, and 0.1 μg/L, respectively.

### 2.2. Microcosm Setup

Both control (biotic) and diesel treatments were set up in triplicate for each time point. Sampling was carried out after incubation for 0, 3, 7, and 21 days. The diesel fuel used in this study was purchased from a local Shell gasoline station. Each microcosm contained 900 mL of water in a 1 L Schott bottle with a Teflon cap. Diesel treatments were amended with diesel fuel at a final concentration of 10 mg/L. The microcosms were incubated on shakers at 25 °C at a speed of 15 rpm under natural light.

### 2.3. Hydrocarbon Extraction and Analysis

Diesel hydrocarbons in each microcosm were solvent–solvent extracted at days 0, 3, 7, and 21 using dichloromethane. Briefly, 50 mL of dichloromethane (≥99.8%) was added to a sterile funnel containing 300 mL of water. Hydrocarbons were extracted by vigorous shaking for ~10 min. The organic phase was collected, and the water was re-extracted twice. The total extracts were concentrated to 10 mL with an IKA RV10 rotary evaporator (IKA, Königswinter, Germany) at 750 pa and 35 °C. The concentrated hydrocarbon extract was purified by passing through a magnesium silicate chromatography column, and eluted with *v*(n-hexane):*v*(dichloromethane) = 1:1. The eluate was concentrated to ~1 mL using the rotary evaporator, and the volume was adjusted to 2 mL with chromatography-*n*-hexane (≥98%, Sigma-Aldrich, Oslo, Norway). *n*-Alkane (C7-C40) and 16 EPA PAHs were quantified by gas chromatography–mass spectrometry (GC–MS) installed with an automatic injector (Agilent 7890-5977MS, Santa Clara, CA, USA). The temperature programs for *n*-alkanes and PAHs were described previously [26]. Quantitative analysis was performed using an eight-point external standard consisting of *n*-alkane (C8-C40, Sigma-Aldrich, Taufkirchen, Germany) or 16 EPA PAHs (Sigma-Aldrich, Germany). The diluted concentrations were 0, 1, 2, 5, 10, 25, 50, and 100 mg/L for both standards. Compound identification was based on individual mass spectra and retention times in comparison to library data and to external standards that were injected and analyzed under the same conditions [27].

### 2.4. Microbial Biomass Production

Microbial biomass production based on ^3^H-leucine incorporation was determined as a general index of microbial activity or growth rates [28]. The ^3^H-leucine incorporation assay was conducted at 25 °C for each microcosm in triplicate (n = 3). For each incubation, 1.5 mL subsamples were amended with 3.5 nM ^3^H-leucine (activity: 0.47 μCi), and then incubated for 2 h at 25 °C. Killed controls were amended with 100% trichloroacetic acid (TCA) prior to adding ^3^H-leucine. The volume ratio of TCA to seawater equaled 1:15. The incubations were terminated using the same procedure by adding TCA. The cells in these samples were washed with 5% TCA and 80% ethanol. Afterwards, 1.75 mL scintillation cocktail (Ultima Gold™, Perkin Elmer, Waltham, MA, USA) was added to the tubes, and the radioactivity was quantified using a Beckman LS-6500 liquid scintillation counter (Beckman, Fullerton, CS, USA). The rate of bacterial production was calculated as described previously [28].

### 2.5. Microbial ^14^C-hydrocarbon Oxidation Rates

^14^C-hexadecane and ^14^C-naphthalene radiotracer assays were applied for all microcosms to monitor the microbial degradation rate of *n*-alkanes and PAHs in water samples, respectively, as described previously [29]. For each sample, 8 mL of water subsample was transferred to a headspace-free scintillation vial, and amended with ^14^C-hexadecane or ^14^C-naphthalene (American Radiolabel Chemicals; ARC). The unit of radioactivity per 8 mL sample was 1.76 nCi. Killed controls were amended with 2 mL 2 M NaOH solution prior to tracer addition. The incubation was also halted by adding 2 mL 2 M NaOH solution. Afterwards, 1 g of activated carbon (Sigma Aldrich, Burlington, MA, USA) was added to the water for absorbing the remaining ^14^C-hexadecane/^14^C-naphthalene. The water was then transferred to a 250 mL flask. A total of 5 mL H_3_PO_4_ (≥80% wt) was added to release the ^14^C-dissovoled inorganic carbon (DIC). The ^14^C-CO_2_ was trapped by Carbo-Sorb (Perkin Elmer, USA) in an acid digestion system as described [29]. The radioactivity measurements were performed with a Beckman LS-6500 liquid scintillation counter (Beckman, Fullerton, CA, USA) with scintillation cocktail (Permafluor^®^ E+, Perkin Elmer, USA). The rate of ^14^C-hexadecane/naphthalene oxidation was calculated as described previously [29].

### 2.6. DNA Extraction and Cell Counting

A total of 400 mL of water was filtered through 0.2 μm filter membranes (Millipore, Burlington, MA, USA), and the membranes were cut into fractions using sterile scissors prior to DNA extraction. The total DNA was extracted from filters using a FastDNA spin kit (Qbiogene, Irvine, CA, USA) according to the instructions of the manufacturer. The quality and purity of the extracted DNA were determined using a NanoDrop spectrophotometer (NanoDrop Technologies, Wilmington, Germany). The DNA was stored at −80 °C until further analyses.

### 2.7. Illumina Miseq Sequencing of 16S rRNA Gene Amplicons and Data Analysis

The composition of the microbial communities was analyzed by sequencing the V4–V5 region of the 16S rRNA gene. All amplifications were performed for each treatment using the 515F/907R primer pair. The MiSeq sequencing data were analyzed using Quantitative Insights Into Microbial Ecology, v2 (QIIME2) software package [30]. The original sequences were demultiplexed using the DADA2 plugin with chimera removal to infer amplicon sequence variants (ASVs) [31], which are high-resolution classifications for microbes analogous to the traditional use of operational taxonomic units [32]. For taxonomic assignment, the 16S rRNA gene sequences were assigned using the QIIME2 “q2-feature-classifier” trained on the Silva database (nr_v132).

### 2.8. Statistical Analyses

The resulting data of isotope assays were analyzed though multiple comparisons using one-way ANOVA analysis followed by LSD Duncan. SPSS version 20.0 was used for these statistical analyses (SPSS Inc., Chicago, IL, USA). A *p*-value significance threshold of 0.05 was employed. Comparisons of bacterial community compositions in different treatments or different time points was performed using one-way analysis of similarities (ANOSIM) using Plymouth routines in multivariate ecological research (PRIMER 6).

## 3. Result

### 3.1. Response of Microbial Total Activities to Diesel Addition

**Figure 1:**
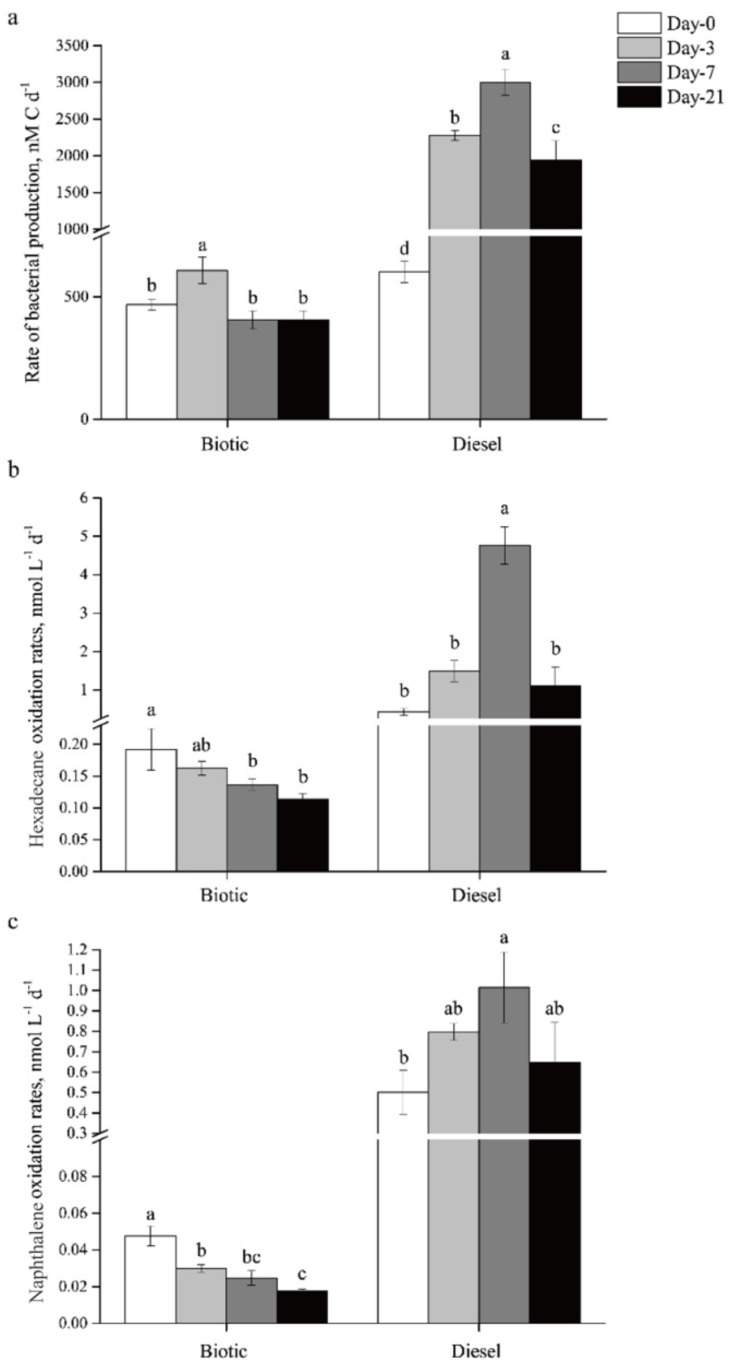
Changes in the rate of bacterial production (**a**), hexadecane oxidation rate (**b**), and naphthalene oxidation rate (**c**) incubated with (diesel) or without (biotic) diesel across the incubation. Different letters above the columns for each treatment indicate a significant difference (*p* < 0.05). Day: 0 d, 3 d, 7 d, and 21 d indicate the microcosms incubated for 0, 3, 7, 21 days, respectively. Error bars represent standards errors of three biological triplicates.

The activities of total microbial communities were determined and monitored using ^3^H-leucine-based radiotracer assays (Figure 1a). The result show that diesel addition significantly enhances microbial activity, as reflected in rates of bacterial production (^3^H-leucine incorporation, *p* < 0.05). The rate of bacterial production increases significantly from 602.0 nmol C L^−1^ d^−1^ at day 0 to 2276.1 and 3002.6 nmol C L^−1^ d^−1^ in diesel treatment after incubation for 3 and 7 days, representing 3.8- and 5.0-fold increases, respectively. The bacterial production rate reaches its maximum at day 7, and then drops down at day 21. The rate at day 21 is still significantly much higher in the diesel treatment than in the biotic control.

### 3.2. Response of Oil-Degrading Microorganisms to Diesel Oil Addition

Radiotracer assays allowed direct quantification of alkane (^14^C-hexadecane) and PAH (^14^C-naphthalene) oxidation rates, and were applied to monitor the changes in microbial oil-degrading activities to diesel addition during the incubation (Figure 1b,c). Similar trends were observed for hexadecane and naphthalene oxidation rate. Both the rates of hexadecane and naphthalene oxidation reach their maximums at day 7 in the diesel treatment, representing 11.0- and 2.0-fold increases compared to day 0, respectively, and then decrease slightly at day 21. The dynamic changes in hexadecane and naphthalene oxidation rates with time is not correlated with that of bacterial production rate in the diesel treatment (*p* > 0.05). The biggest increase in the bacterial production rate occurs at day 3, whereas the rates of hexadecane and naphthalene oxidation do not increase significantly until day 7. The biggest increase in the hexadecane oxidation rate occurs during the incubation between day 3 and day 7.

It is also noteworthy that the response of oil-degrading bacteria to diesel addition is much faster than that of the total microbial community. Compared to the biotic controls without diesel addition, in less than about 24 h after the addition of diesel (the necessary incubation time in radiotracer assays), the oxidation rates of hexadecane and naphthalene suddenly increase by 2.3 and 10.5 times at day 0, respectively. However, such a sudden increase is not observed in the rates of bacterial production.

### 3.3. The Degradation of Total n-Alkanes and PAHs during the Incubation

**Figure 2:**
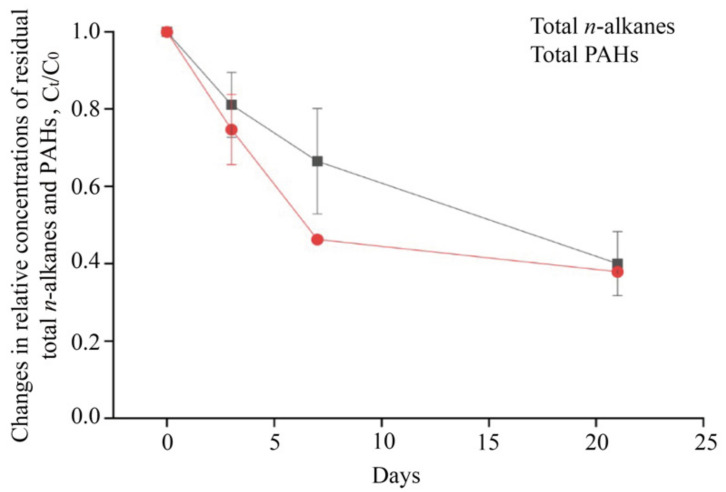
Changes in relative concentrations of residual total *n*-alkanes and PAHs incubation with diesel across the incubation in the diesel treatment. C_t_/C_0_ represent the relative concentrations of total *n*-alkanes and PAHs, respectively, at different time point (C_t_) compared to their concentrations determined at day 0 (C_0_).

Degradation of the total *n*-alkanes and PAHs over the incubation were evaluated using the relative concentration of the total *n*-alkanes and total PAHs at different time points to day 0 (Figure 2). The result shows that the degradation ratio of total dissolved *n*-alkanes and PAHs decreases as time progressed. By day 3, the degradation ratio of the total *n*-alkanes and PAHs decreases to 81.1% and 74.7% of the initial concentrations at day 0, respectively. At day 7, the ratio of degraded PAHs (53.8%) is remarkably higher than that for total alkanes (33.5%). However, the degradation ratio of total PAHs and alkanes is similar at day 21. The *n*-alkanes and PAHs may also undergo several abiotic dissipation mechanisms, such as volatilization and photodegradation.

### 3.4. Shift in the Microbial Community Composition and Key Potential Oil-Degrading Bacteria during the Incubation

**Figure 3:**
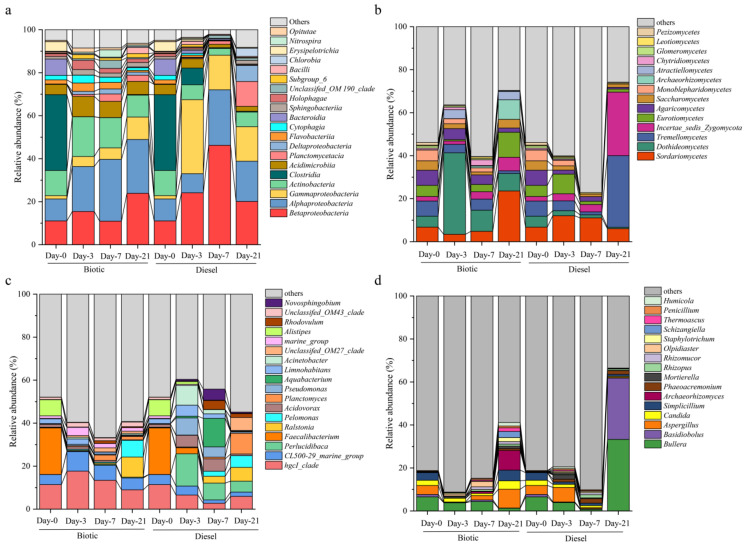
Community composition of bacteria at class (**a**) and genus (**c**) levels and fungi at class (**b**) and genus levels (**d**) in the treatments with (diesel) or without (biotic) diesel. The graphs only present the classes or genera whose relative abundance are >0.2% and >0.01%, respectively. The remaining classes or genera are presented as “others”.

To reveal the response of microbial communities to diesel addition, the successional pattern of microbial was monitored over time. The result reveals that the addition of diesel significantly shifts the structure of microbial communities, and the composition of microbial communities varies at different sampling days (Figure 3). At the class level, *Gammaproteobacteria* and *Betaproteobacteria* are significantly stimulated in the diesel treatment. Different specific bacterial genera are preferentially selected at different time point, underscoring the role of diesel in driving variation in diesel-degrading groups. After 3 days, the relative abundance of *Perlucidibaca*, *Acinetobacter*, *Pseudomonas*, *Acidovorax*, and *Limnohabitans*, increases from 0.1%, 0.2%, 0.2%, 1.3%, and 2.2% at day 0 to 15.0%, 9.2%, 8.0%, 5.4%, and 5.1%, representing 230.8, 60.5, 34.2, 4.1, and 2.3-fold increases, respectively. By contrast, the dominate bacterial genera are replaced by genera *Aquabacterium*, *Novosphingobium*, and *Rhodovulum* at day 7, which increase by 1021.2, 59.6, and 60.7-fold, respectively, compared to day 0. At day 21, the dominant genera shift to *Ralstonia*, *Pelomonas*, *Planctomyce,* and OM27_clade, which account for 9.1%, 5.5%, 9.6%, and 6.0% of the total bacterial community, respectively.

Members of the fungal class *Sordariomycetes*, *Incertae_sedis_Zygomycota*, *Eurotiomycetes,* and *Tremellomycetes* increase over the 21 day microcosm incubation. The fungal response closely resembles the successional patterns at the genus level in the diesel treatment. Among the main responders, *Mortierella* (2.0%), *Aspergillus* (6.8%), and *Thermoascus* (0.6%) reach their maximum relative abundance at day 3, whereas *Phaeoacremonium* (2.0%), *Rhizopus* (1.5%), and *Staphylotrichum* (0.6%) are the dominant genera at day 7. Notably, the relative abundance of *Bullera* (33.2%) and *Basidiobolus* (28.5%) at day 21 increases substantially by 5.1 and 25.8-fold, respectively, compared with those at day 0.

## 4. Discussion

### 4.1. Fast Responses of Microbial Activities and Diesel-Degrading Microorganisms to Diesel in the River Ecosystem

The increased activities of the whole microbial communities and diesel-degrading microorganisms indicate that diesel degraders in the river water are primed for anthropogenic sources of hydrocarbons. The fast response of the microbial community to the amendment of diesel could be due to the sudden increase in the concentration of organic carbon. Prior studies about microbial activity impacts found that specific bacterial metabolites, such as transparent exopolymer particles formation, and exoenzyme activities (e.g., glucosidase, peptidase, lipase), were fueled by oil additions [33,34]. The increased bacterial protein synthesis rate in the diesel treatment suggests the potential diesel microbial degradation. The fast buildup of diesel hydrocarbon oxidation activities is demonstrated by the sudden increases in ^14^C-hexadecane- and ^14^C-naphthalene-oxidizing rates immediately after the setup of the experiments (<24 h, the incubation time of ^14^C-radiotracer assay). This result is probably due to the fast induction of enzymes involved in the biodegradation and metabolism of diesel compounds [35,36], as the activation of enzymes generally happens in minutes or hours [37]. In addition, the inconsistencies of the whole microbial production rates and the ^14^C-hexadecane/^14^C-naphthalene oxidation rates indicate that, instead of all the microbial individuals, it is probably the diesel-degrading microorganisms that instantly respond to the diesel addition. This result agrees with previous studies reporting that the addition of oil to water led to an immediate bloom of oil-degrading microorganisms and a rapid rate of oil degradation [38,39].

The activities of microbial oil biodegradation were time- and compound-dependent. The biodegradation rates increase gently in the initial 3 day incubation, and reach the maximal rate at day 7, and decrease slightly at day 21. The changes in biodegradation rates also couple well with the decrease in total *n*-alkane and PAHs. This phenomenon is likely a result of a combination of biological and chemical factors. Fast-growing bacteria generally utilize short-chain alkanes or benzenes rapidly responded to oil spills [14]. This finding is supported by the much higher microbial oxidation rate of ^14^C-hexadecane compared to that of ^14^C-naphthalene (Figure 1b,c). With the progress of oil biodegradation, the diesel hydrocarbons in the water phase are consumed by microbial degradation activities, and the newly and more diverse dissolved oil hydrocarbons are continuously trapped in to the water phase through the biosurfactants-assisted emulsification [40,41], or chemical dissolution equilibrium [42]. This could further stimulate the activity of diesel-degrading microorganisms, which explains the highest oil biodegradation rate at day 7. Previous studies indicated that oil addition initially promoted the growth of some microbial populations that increased the bioavailability of hydrocarbons prior to the initiation of insoluble-hydrocarbon degradation, or induced cometabolic enzymes [43,44]. The decrease in the ^14^C-hexadecane and^14^C-naphthalene oxidation rate at day 21 could be due to nutrient limitations, such as N or P [45], or the non-bioavailable oil hydrocarbons [46].

It is noteworthy that the higher multiple to which the increased ^14^C-napthalene oxidation rate was stimulated by the diesel addition was observed compared to the ^14^C-hexadecane. This seems to contradict to the previous finding that generally linear alkanes or low-molecular-weight hydrocarbons are preferentially degraded by oil-degrading microorganisms, followed by PAHs or high-molecular-weight hydrocarbons [14,47]. Two reasons might explain this observation. Firstly, the solubility of hexadecane is several orders lower than that of naphthalene [48]. The higher bioavailability of naphthalene could support the faster increase in naphthalene oxidation rate. Secondly, the efficient PAHs-degrading microbial group might also contribute to the sharp increase in naphthalene oxidation activity. The high naphthalene degradation-related gene and naphthalene exposure of the water in our previous study also supports this deduction [27].

In addition, river fresh water has much higher hydrocarbon degradation rates than those in marine environments, inferred from the data of the radiotracer assay. For instance, the hexadecane oxidation rates in the river water sample (1.49–4.76 nmol/L/d) is of the same magnitude as those detected in other river water samples (5.40–21.8 nmol/L/d) [49], but much lower than in marine environments (0.0005–0.01 nmol/L/d), such as offshore seawater [21], and deep-sea water [34].

### 4.2. The Enrichment and Succession of Diesel-Degrading Bacteria and Fungi during the Incubation

The addition of diesel alters the microbial community and triggers specific microbial genera. The successive patterns of diesel-degrading bacteria and fungi observed are likely a result of a combination of the dynamically changing oil components and nutrients available for oil-degrading microorganisms [10,14]. The proliferation of hydrocarbons *Perlucidibaca*, *Acinetobacter*, *Pseudomonas*, and *Acidovorax* highlight the importance of these taxonomic groups as being central to diesel degradation in the initial period, with highest levels reached at day 3. Members of *Perlucidibaca* have previously been identified as a part of hydrocarbon-degrading communities in seawater [50], and rarely detected in freshwater. However, the capacity of *Perlucidibaca* utilizing *n*-alkanes has been well documented [50,51]. *Acidovorax* and *Acinetobacter* are reported for their almost exclusive preference for aliphatic hydrocarbons and for commonly blooming soon after oil is introduced to an environment [52,53]. *Pseudomonas*, which are known for degrading different types of hydrocarbon mixtures [54], was highly enriched in the diesel treatment. *Pseudomonas* sp. has been observed as a main oil hydrocarbon responder in fresh water from lake wetlands [55] and rivers [56]. At day 7, the relative abundance of these genera slightly decreases and they are outperformed by *Aquabacterium*, which is the most abundant genus. *Aquabacterium* has been identified as an oil degrader [57], and was strongly stimulated by diesel in a previous incubation study [58]. Another enriched genus at day 7, *Novosphingobium*, was also observed in high abundance in a diesel-related microbiology study [59]. *Novosphingobium* sp. and another stimulated genus *Rhodovulum* are generally associated with several PAHs biodegradation [60,61], which may also explain the significant increased PAHs biodegradation ratio at day 7. At day 21, the potential of the dominant bacterial genera by common and widespread OM27_clade [62], *Planctomyces* [63], and *Ralstonia* [64] suggests that the microbial community changes from indigenous oil-degrading bacteria to more general heterotrophic bacteria during the later period of oil spills. Members of *Ralstonia* and *Planctomyces* may utilize diesel-derived organic intermediates produced by hydrocarbon degraders [64,65].

Fungi were implicated in diesel hydrocarbon degradation, as shown by the dynamic change in fungal community structures over the incubation. The potential of fungi to remove oil pollutants was described previously [20]. At day 3, the dominant diesel responder *Aspergillus* was frequently isolated from oil-contaminated environments and showed great capacity in degrading diesel hydrocarbons [19,66]. The extracellular enzymes and biosurfactant produced by some *Aspergillus* spp. cultures can efficiently degrade alkane, aromatic, resins, asphaltene, and disperse oil hydrocarbons [67]. The increased concentrations of bioavailable hydrocarbons by the fungal biosurfactants could further stimulate bacterial hydrocarbon degradation [68]. Another enriched fungal genus in the diesel treatment was *Mortierella*, which was also reported as a PAH degrader that can cooperate with bacteria and stimulate PAHs degradation [69]. At day 7, the dominant fungi became overprinted by *Phaeoacremonium*, which was previously detected in an oil biodegradation study [70]. *Bullera* and *Basidiobolus* are known for their organic decomposition abilities and it is possible that their low specificity of substrate metabolism allowed them to degrade various diesel biodegradation intermediates (i.e., peptide- and carbohydrate-rich organic macromolecules) and outperform other diesel-degrading fungi at day 21 [71,72]. These results suggest that fungi should not be ignored in developing effective strategies of oil bioremediation.

## 5. Conclusions

Our results demonstrate that diesel addition enhances both microbial diesel-degrading activities and microbial biomass production activities, and there is a differential distribution of bacterial and fungal communities in river water amended with diesel. A rapid microbial degradation activity of oil components was induced within 24 h in the river water. Over time, the hexadecane and naphthalene microbial oxidation rates, and microbial structure succession patterns change and are triggered by the addition of diesel. The succession patterns may be due to multiple reasons, including the bioavailability of diesel compounds and the specialized degradation of different diesel hydrocarbons by different microbial groups. Several common taxonomic genera that are known obligate and generalist hydrocarbon degraders were observed in abundance in the diesel treatment, especially in the early days of the incubation period, such as bacterial genera *Acinetobacter*, *Perlucidibaca*, *Pseudomonas*, and *Acidovorax*, and fungal genera *Aspergillus* and *Mortierella*. The response of bacterial and fungal communities to diesel addition also highlights that the interaction among fungi and bacteria in the oil biodegradation process warrants further research. Overall, this study suggests that the progression of microbial diesel degradation could be experimentally predicted and serves as references for future river oil bioremediation strategies.

## Data Availability

The data presented in this study are available on request from the corresponding author.

## Supplementary Materials

The following supporting information can be downloaded at: https://www.mdpi.com/article/10.3390/microorganisms11030698/s1, Figure S1: Map and photographs showing the location and surrounding environment of the sampling site in urban section (Nanchong) of the Jialing River.
