# Supplementary material for: Succession Patterns of Microbial Composition and Activity following the Diesel Spill in an Urban River
Source: Microorganisms. 2023 Mar 8;11(3):698. doi: 10.3390/microorganisms11030698 (PMC10058704; doi:10.3390/microorganisms11030698)
Supplement: Supplementary file 1 [file microorganisms-11-00698-s001.zip › microorganisms-2254412-supplementary.pdf]

Supplementary Material

Succession patterns of microbial composition and activity following the diesel spill in an urban river

Ruiyu Yang, Chao Peng, Yuqiu Ye, Yun Tang, Lu Lu

This file includes:

Total Number of Figures: (1)

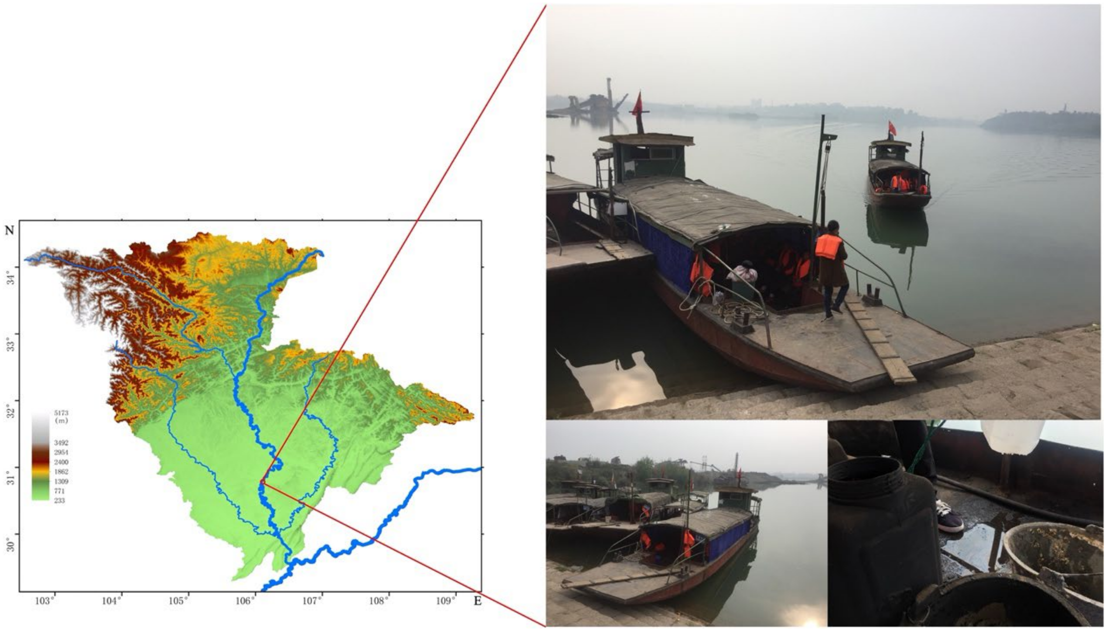

Figure S1. Map and photographs showing the location and surrounding environment of the sampling site in urban section (Nanchong) of the Jialing River.
